# RBM5 inhibits tumorigenesis of gliomas through inhibition of Wnt/β-catenin signaling and induction of apoptosis

**DOI:** 10.1186/s12957-016-1084-1

**Published:** 2017-01-06

**Authors:** Yuanpei Jiang, Hongling Sheng, Lei Meng, Hongsheng Yue, Bo Li, Aijun Zhang, Yanan Dong, Yuguang Liu

**Affiliations:** 1 Department of Neurosurgery, Ji’nan Central Hospital Affiliated to Shandong University, Liberate Road No. 105, Ji’nan, 250013 China; 2 Department of Pediatrics, Shandong Jiaotong Hospital, Central Wuying Hill Road No. 12, Ji’nan, 250031 China; 3 Department of Neurosurgery, Qilu Hospital affiliated to Shandong University, Cultural West Road No. 107, Ji’nan, 250012 China

**Keywords:** Apoptosis, Caspase3, Gliomas, RBM5, Wnt/β-catenin signaling

## Abstract

**Background:**

Gliomas are one of the most common malignant brain tumors and bring a big threat to human life as traditional therapy is unsatisfactory. RBM5 was a RNA-binding motif protein and was reported as a tumor suppressor. But the role of RBM5 in gliomas was unknown.

**Methods:**

The mRNA level of RBM5 was determined in gliomas tissues and cell lines by real-time quantitative PCR (qRT-PCR) assay while the association of RBM5 expression with prognosis was analyzed by Kaplan-Meier method and compared by log-rank test. Lentivirus was used to overexpress RBM5 in gliomas cells. MTT and BrdU incorporation assay were used to determine cell proliferation and DNA synthesis when the ability of cell migration and invasion was analyzed by transwell assay with/without Matrigel. Cell apoptosis rate was determined with fluorescence-activated cell sorting (FACS) method. Then, expression of apoptosis molecules and critical members in Wnt/β-catenin pathway were detected by western blot analysis.

**Results:**

RBM5 was shown to be downregulated in gliomas tissues and gliomas cell lines. And decreased RBM5 expression was clinically correlated with tumor stage, patient age, and poor prognosis of gliomas patients. The proliferation and DNA synthesis was dramatically inhibited when RBM5 was overexpressed in SHG44 or U251 cells. Also, the ability of cell migration and invasion was disrupted. Then, the level of β-catenin and Cyclin D1 significantly decreased when DKK1 and P-GSK-3β increased reversely in SHG44 cells, which suggested that RBM5 inhibited canonical Wnt/β-catenin signaling. Meanwhile, we demonstrated that caspase3-mediated apoptotic pathway was activated by RBM5 as Bax, TNF-α, and cleaved caspase3 were greatly upregulated while antiapoptotic molecule Bcl-2 was downregulated. Additionally, that apoptotic rate increased significantly from less than 1 to 32% in RBM5-overexpressed SHG44 cells further supported the pro-apoptosis role of RBM5 in gliomas cells.

**Conclusions:**

RBM5 plays a suppressor role in human gliomas by inhibiting Wnt/β-catenin signaling and inducing cell apoptosis. This study improves our knowledge about the carcinogenesis and progression of human gliomas, which would greatly contribute to the therapy for gliomas patients.

## Background

Gliomas are one of the most common malignant brain tumors and account for about 80% of all primary malignant central nervous system (CNS) tumors [1]. The latest statistics data predict that about 23,770 new cases and 16,050 new deaths of CNS tumors would occur in 2016 in the USA [2]. What is worse, the outcomes of patients with the gliomas particularly with the glioblastoma were very disappointing despite the great advancement in therapeutics including surgical remove, chemotherapy, and radiation therapy. The median survival time of patients diagnosed with glioblastoma ranged from 12 to 15 months while the 5-year survival rate was less than 5% [3]. So, it is urgent to develop novel therapeutics to fight against gliomas and to improve the survival of gliomas patients.

Elucidation of molecular mechanism underpinning tumorigenesis and progression has been a promising way to develop novel therapeutics. For example, the study about the critical role of VEGF during tumor angiogenesis has directly prompted the come-out of avastin against NSCLC [4]. The important roles of tyrosine kinase in cell proliferation caused synthesis of a series of tyrosine kinase inhibitors [5]. RNA-binding motif protein 5 (RBM5), also called LUCA-15 or H37, is a nuclear RNA-binding protein which contains two RNA recognition motifs, two zinc fingers and a serine-arginine-rich domain [6]. It is located at chromosomal locus 3p21.3 which domain was reported to be the most frequently deleted or mutated in a series of human cancers [7]. For example, RBM5 was often genetically deleted in lung, renal, and breast cancer [8]. Moreover, RBM5 was evaluated as a therapeutic and diagnostic marker in lung cancer [9]. Overexpression of RBM5 inhibited growth of human prostate cancer while it was demonstrated to be downregulated in serous ovarian carcinoma [10, 11]. In fact, accumulating data suggest that RBM5 was a tumor suppressor and inhibited tumor growth or progression by triggering apoptosis and inducing cell cycle G1/S arrest [12]. In early research, RBM5 was reported to regulate the splicing of an apoptotic factor Fas and resensitize lung cancer cells A549 to cisplatin [13]. Also, RBM5 was shown to inhibit Wnt/β-catenin signaling in A549 cells [14]. Wnt/β-catenin signaling was vital to cancer cell proliferation. However, there was no report about the role of RBM5 in gliomas.

In this study, we found RBM5 was decreased in gliomas tissue samples and clinically correlated with poor prognosis in gliomas. To investigate the role of RBM5 in gliomas as well as the mechanism mediated by RBM5, a series of in vitro experiments was designed. And we showed that RBM5 played a suppressor role in gliomas by inhibiting canonical Wnt signaling and enhancing apoptosis.

## Methods

### Cell lines and sample tissues

Cell lines (SHG44, U87, U251) were obtained from Cell Bank Type Culture Collection of Chinese Academy of Sciences (CBTCCCAS, Shanghai, China) and maintained in DMEM (Gibco, USA) containing 10% fetal bovine serum, 100 units/mL penicillin, and 100 μg/mL streptomycin at 37 °C in a 5% CO2 incubator.

A total of 51 frozen gliomas tissues were obtained from Qilu Hospital of Shandong University. The diagnosis and classification were based on histological examination according to the WHO criteria. Written informed consent was obtained from all patients. The experimental protocols were approved by the Ethics Committee of Qilu Hospital of Shandong University. All experiments comply with the laws in China.

### Quantitative real-time PCR (qRT-PCR)

Total RNA from tissues or cell lines were extracted using Trizol (Invitrogen, USA) and treated with RNase-free DNase (Promega, USA). About 2 μg total RNA was used to synthesize cDNA with first strand cDNA synthesis kit (Fermentas, USA). Then, qRT-PCR analysis was performed on the Applied Biosystems 7500 Fast Real-Time PCR system and software according to the manufacturer’s protocol. Relative expression (RE) of RBM5 was calculated with the listed formula:

$$ \mathrm{R}\mathrm{E}={2}^{-\left\{{}^{\varDelta \mathrm{C}\mathrm{t}\left(\mathrm{sample}\right)\ \hbox{-}\ \Delta \mathrm{C}\mathrm{t}\left(\mathrm{control}\right)}\right\}} $$

where ∆Ct = Ct (RBM5) − Ct (GAPDH). All samples were repeated in triplicates.

### Production of lentivirus and infection of cell lines

pLVX-Puro plasmid was used to express RBM5 (NM_005778.3). The coding sequence of RBM5 was cloned into empty pLVX-Puro. Then, modified plasmid were co-transfected into 293 T cells with lentiviral packaging plasmids to generate lentivirus vector expressing RBM5. About 5 × 10^5^ glioma cells/well were plated in six-well plates and treated with lentivirus vector at mol 10 when cells grown to 40% confluence. After infection for 72 h, the expression efficiency of RBM5 was determined with qRT-PCR or western blot analysis.

### MTT proliferation assay

Gliomas cells expressing RBM5 were seeded into a 96-well plate at 3 × 10^3^cells/well in 100 μl DMEM (Gibco, USA). About 20 μl of MTT solution (Sigma, 5 mg/ml) was added into each well for 5 days, and plates were incubated for another 4 h at 37 °C before. Then, 200 μl of dimethyl sulfoxide (DMSO; Sigma) was added to dissolve the crystals. Absorbance values (A) at 490 nm were measured on a microplate reader. Each experiment was repeated in triplicates.

### BrdU incorporation assay

DNA synthesis in gliomas cells was detected by BrdU incorporation rate. About 5000 cells were seeded into a 96-well plate and incubated for 24 or 48 h. Ten microliters 1 × BrdU were added to the plate from 2 to 24 h followed by adding 100 μl fixing solution for 30 min. Then, cells were washed with wash buffer and incubated with 50 μl 1 × BrdU antibody for 1 h followed by adding of 50 μl 1 × goat anti-mouse IgG, 50 μl TMB substrate solution sequentially. After incubation for 30 min, stop solution was added and absorbance value at 450 nm was measured on a plate reader.

### Transwell assay

The invasive ability of cell lines was analyzed by transwell assay using a transwell chamber with or without a Matrigel-coated filer. About 5 × 10^5^ cells were seeded into the upper chamber with serum-free medium while DMEM with 20% FBS was added into the lower chamber. After incubation for 24 h, cells on the upper surface of the filter were removed and the cells on the lower surface were stained with crystal violet. The number of transferred cells was determined by in five random selected fields per well.

### Apoptosis assay

About 1 × 10^6^ cells were centrifuged for 5 min at 1500 rpm, washed with cold Biolegend’s Cell Staining Buffer buffer, and resuspended in Annexin V Binding Buffer. About 100 μL of cell suspension was transferred into a 5-ml test tube followed by adding of 5 μL of FITC Annexin V and 5 μL of 7-ADD Viability Staining Solution. Gently vortex the cells and incubated at 25 °C in the dark for 15 min, the volume was raised to 500 μL with Annexin V Buffer and analyzed on FACSCanto II Flow cytometry b (BD Biosciences, USA).

### Western blot analysis

Cells infected with lenti-RBM5 were seeded into six-well plates, and total protein was extracted after 72 h with CelLytic™ MEM Protein Extraction Kit (Sigma-Aldrich, USA) followed by quantified with bicinchoninic acid assay (BCA Protein Assay Kit, Generay, Shanghai, China). Then, 5 μg total protein were separated on SDS-polyacrylamide gel, transferred to nitrocellulose membranes, and incubated overnight with primary antibodies against Bcl-2, Bax, β-catenin, cyclin D1 (Santa Cruz Biotechnology, USA) and TCF, DKK1, GAPDH, Caspase-3, TNF-α (Abcam, USA) at 4 °C. After that, membranes were washed and incubated in horseradish peroxidase-conjugated secondary antibody for about 1 h at room temperature and target protein were determined with ECL kit (Pierce, USA).

### Statistical analysis

All data were analyzed by a two-tailed Student’s *t* test for statistical difference with SPSS16.0 based on three independent experiments. The correlation of RBM5 with clinicopathological factors was analyzed by chi-square est. Survival curves were plotted by Kaplan-Meier method and compared by log-rank test. *P* < 0.05 was recorded as significant difference.

## Results

### RBM5 was downregulated in gliomas tissues and correlated with a poor prognosis

**Fig. 1:**
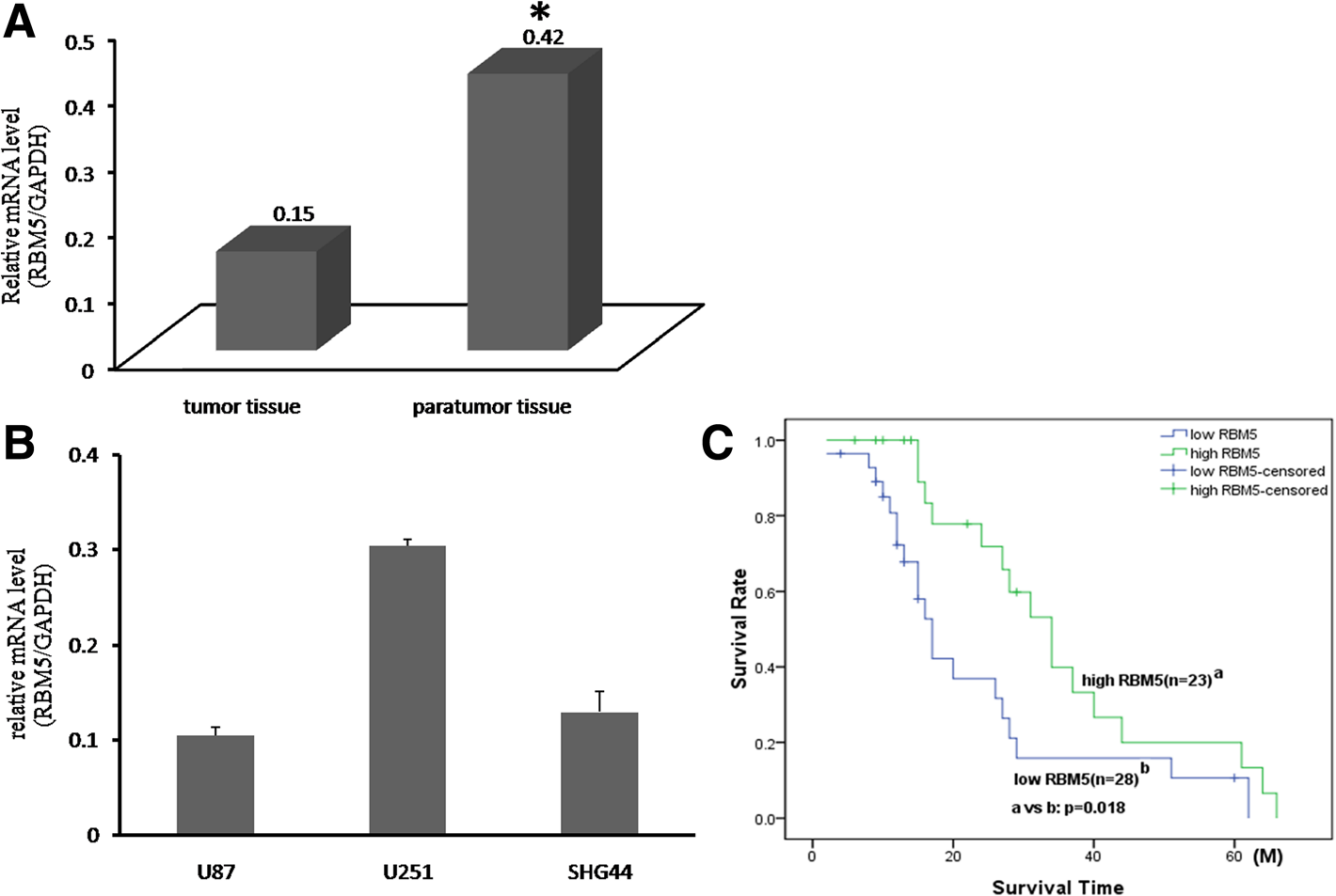
RBM5 was downregulated in gliomas and associated with prognosis of gliomas patients. The expression of RBM5 was detected in 51 gliomas tissues and 3 cell lines by RT-qPCR. Then, the relationship of RBM5 level with survival time was analyzed by Kaplan-Meier method and compared by log-rank test. **a** RT-qPCR analysis of RBM5 mRNA in tumor tissues. **b** RT-qPCR analysis of RBM5 mRNA in cell lines U87, U251, and SHG44. **c** The correlation analysis of RBM5 expression with 5-year survival time in gliomas patients. Data were shown as means ± SD of three independent experiments. *P < 0.05 was considered as statistically significant

To investigate the clinical significance of RBM5 in gliomas, the mRNA level of RBM5 in tumor tissues from 51 patients diagnosed with gliomas and in gliomas cell lines were detected by qRT-PCR assay. It was shown that RBM5 was dramatically reduced in tumor tissues compared to paratumor tissues (Fig. 1a). Also, RBM5 was expressed weakly in three gliomas cells including U87, U251, and SHG44 (Fig. 1b). Then, clinicopathological analysis indicated that downregulated RBM5 was significantly correlated with tumor stage (*P* = 0.004) but not with age (*P* = 0.068) or sex (*P* = 0.405) (Table 1). Moreover, weak RBM5 expression was demonstrated to be associated with poor prognosis. The estimated 5-year survival rate in patients with low RBM5 expression was about 40.5% (*n* = 39), but it was 63.4% in those with high RBM5 expression (*n* = 19). There was a significant difference between these two groups (*P* = 0.018) (Fig. 1c). Our data indicate that RBM5 may function as a suppressor in gliomas.

**Table 1:** RBM5 expression was correlated with clinicopathological characteristics of gliomas

Characteristics	Total	RBM5 expression		*P* value
		Low	High	
All cases	51	28	23	
Age (year)				0.068
<60	22	13	9	
≥60	29	15	14	
Grade	21	9	12	0.148
G1/G2	21	9	12	
G3/G4	30	19	11	
TNM stage				0.004
Early (I/II)	23	8	17	
Late (III/IV)	28	20	8	
Sex				0.405
Male	32	19	13	
Female	19	9	10	

### RBM5 significantly suppressed growth of human gliomas cells in vitro

**Fig. 2:**
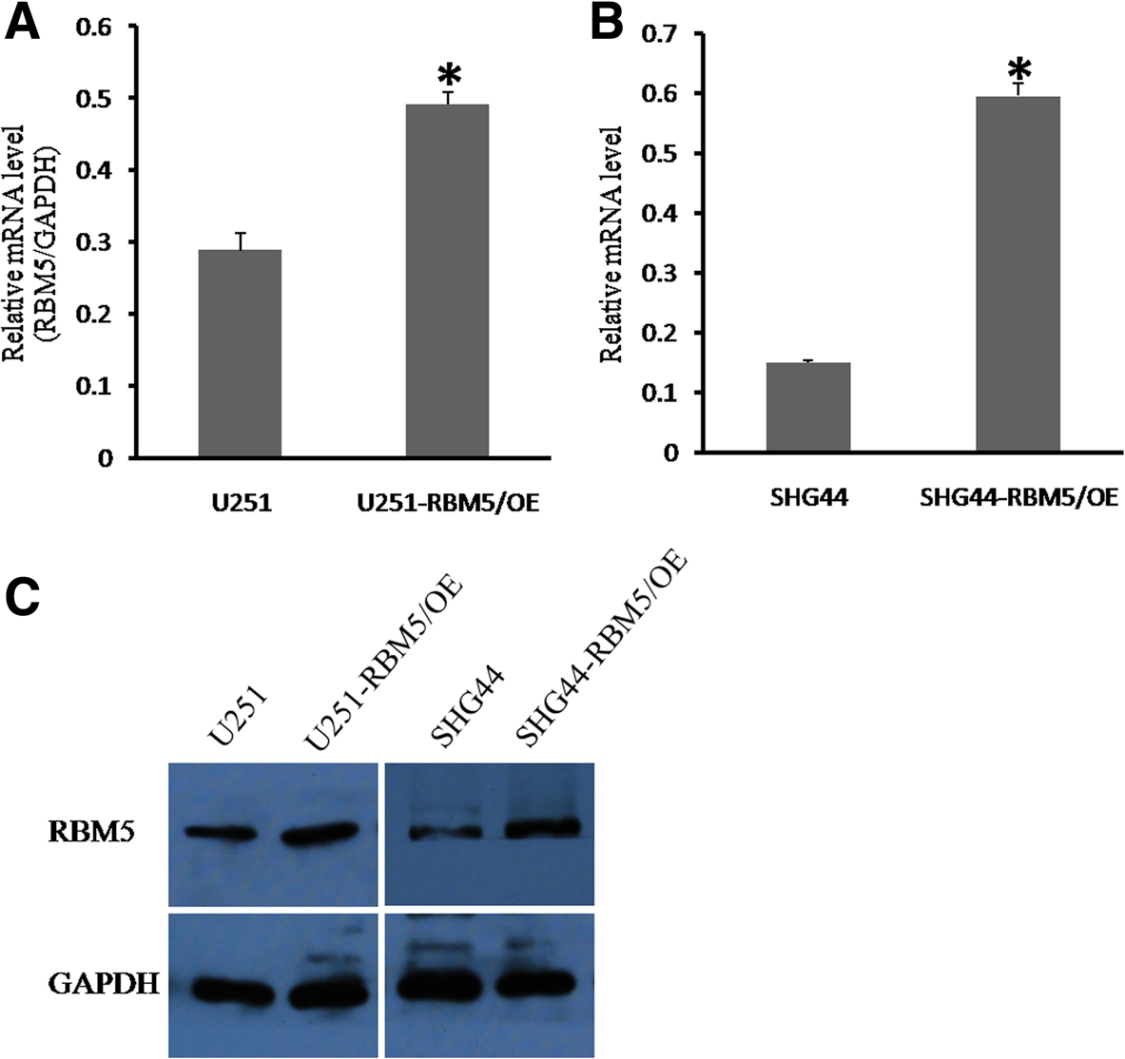
RBM5 was successfully overexpressed in U251 and SHG44 cells. RBM5 was introduced into U251 and SHG44 cells by lentivirus and confirmed by RT-qPCR and western blot analysis. **a** RT-qPCR analysis of RBM5 mRNA in U251 cells. **b** RT-qPCR analysis of RBM5 mRNA in SHG44 cells. **c** Western blot analysis of RBM5 protein in U251 and SHG44 cells. Data were shown as means ± SD of three independent experiments. *P < 0.05 was considered as statistically significant

**Fig. 3:**
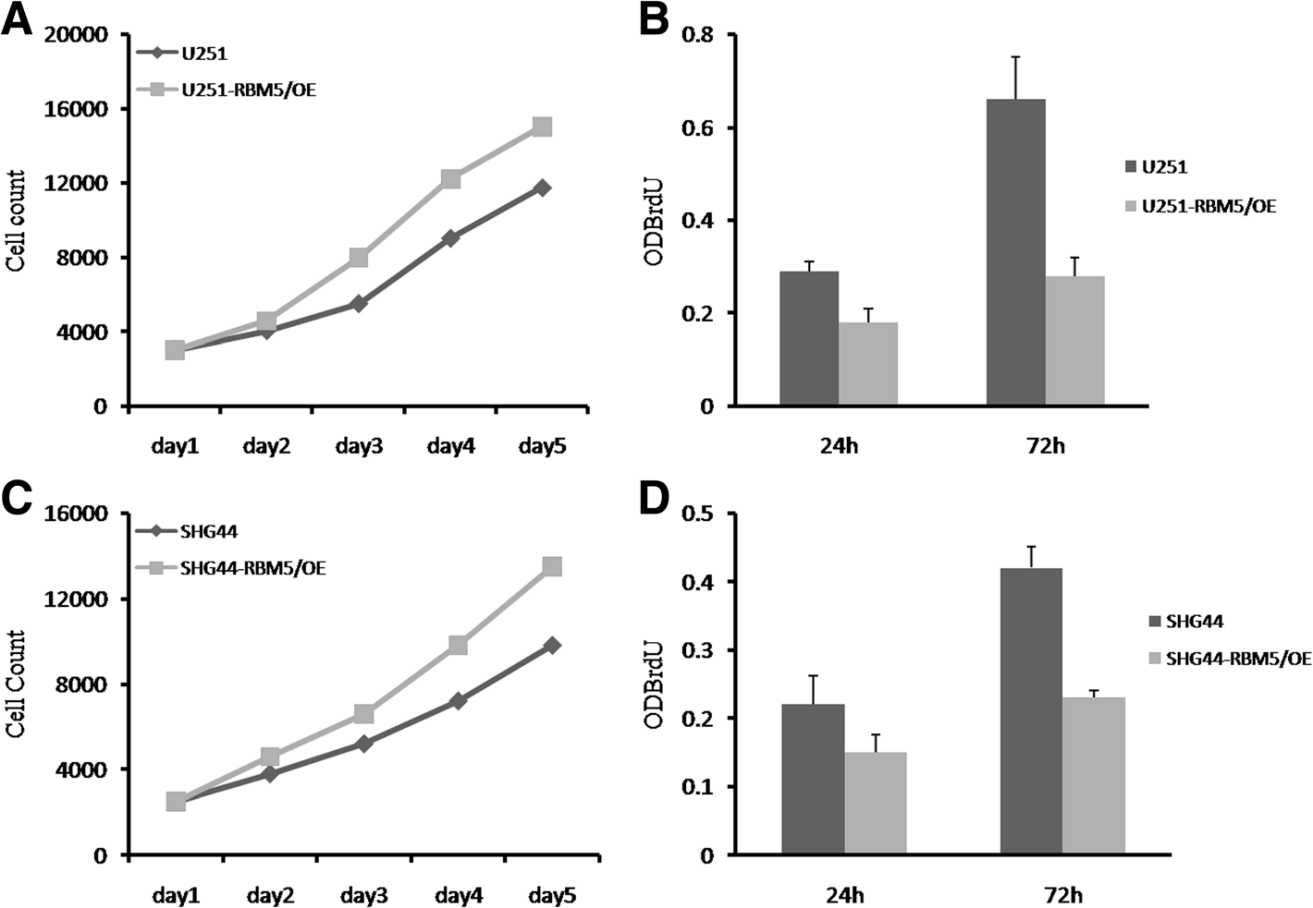
RBM5 inhibited proliferation and DNA synthesis in gliomas cells. Cells expressing RBM5 were seeded into 96-well plates and were counted for consecutive 5 days. To determine DNA synthesis, BrdU was added 2 h after cells were cultured and absorbance value at 450 nm was detected at 24 and 72 h, respectively. **a** The growth of U251 cells overexpressing RBM5. **b** DNA synthesis in U251 cells overexpressing RBM5. **c** The growth of SHG44 cells overexpressing RBM5. **d** DNA synthesis in SHG44 cells overexpressing RBM5. Each experiment was repeated in triplicates. *P < 0.05 was considered as statistically significant

To examine the role of RBM5 in gliomas, RBM5 was overexpressed in U251 and SHG44 cells by lentivirus infection. As shown in Fig. 2, both mRNA and protein level of RBM5 was successfully upregulated in U251 and SHG44 cells compared to the parent cells after lentivirus infection for 96 h. Then, MTT assay and BrdU incorporation assay were employed to determine cell growth rate. It was demonstrated that RBM5 overexpression remarkably reduced the proliferation of both U251 and SHG44 cells (Fig. 3a, c). The proliferation rate at the fifth day was only 22.7% in U251 cells and 30.4% in SHG44 cells compared to the control cells. Similar results were obtained in BrdU incorporation assay in which U251-RBM5/OE cells showed a reduction of 37% BrdU incorporation rate at 24 h and 57% at 72 h (Fig. 3b, d). So, it was conceived that RBM5 potentially delayed the growth of gliomas cells and inhibited the DNA synthesis.

### RBM5 disrupted the migration and invasive ability of human gliomas cells

**Fig. 4:**
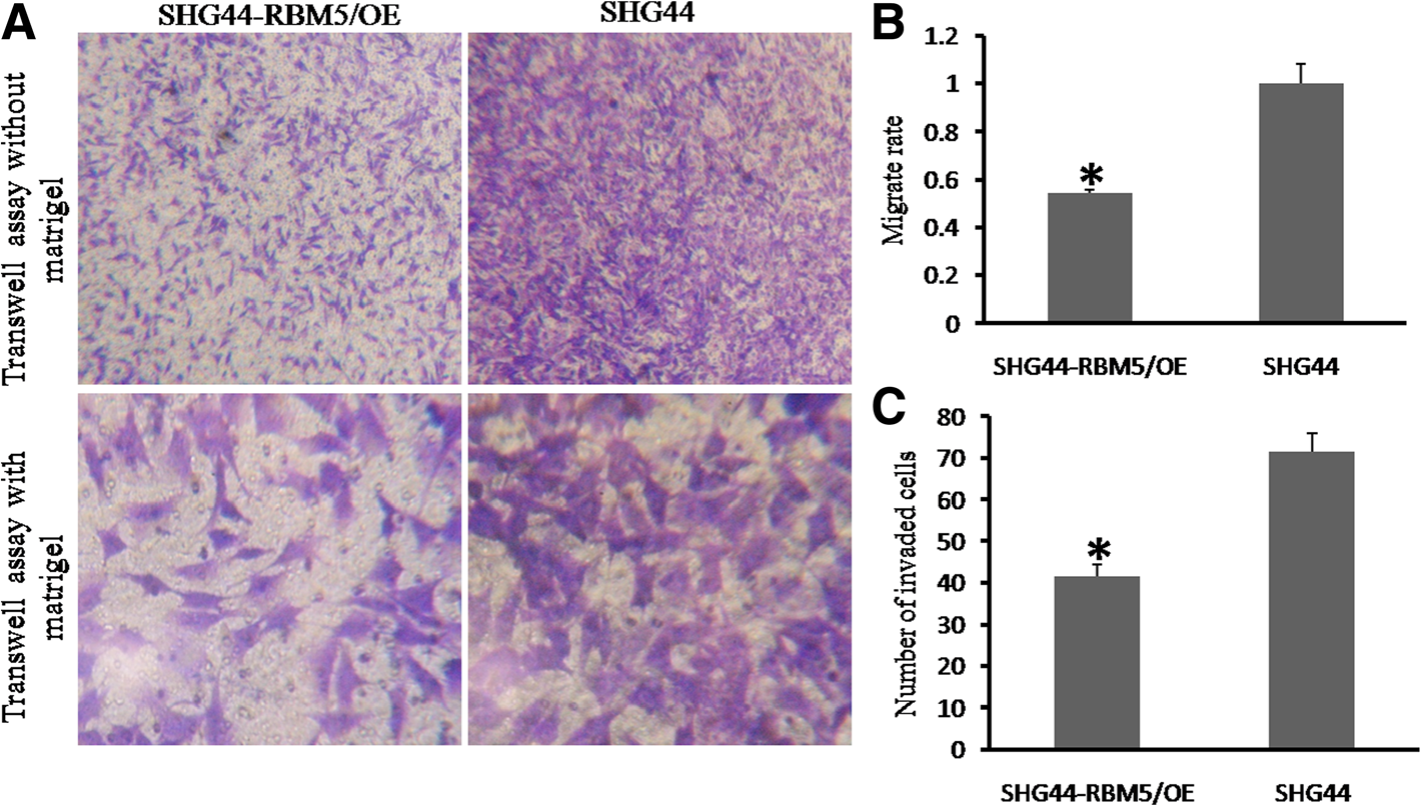
RBM5 disrupted migration and invasive ability in gliomas cells. Cells were seeded into the upper chamber, and the migrated or invasive ability was observed at 24 h in a transwell assay with/without Matrigel. **a** Migration of SHG44 cells was blocked when invasive cells were reduced by RBM5. **b** The migrate rate of SHG44 cells in a transwell assay without Matrigel. **c** The invaded SHG44 cells in a transwell assay with Matrigel. Each experiment was repeated in triplicates. *P < 0.05 was considered as statistically significant

RBM5 was reported to be associated with tumor metastasis [15]. Cell migration and invasion were essential for tumor metastasis. Accordingly, we determined the effect of RBM5 overexpression on migration and invasiveness of gliomas cells. In the transwell assay without Matrigel, we found that RBM5 overexpression significantly blocked the migration of SHG44 cells (Fig. 4a, b). The cells migrated through the filter in RBM5 overexpressed group was about 55% of that in control group. The invasive capacity was also disrupted in RBM5 overexpressed cells. In the transwell assay with Matrigel, the cells transferred through the filter monolayer in SHG44-RBM5/OE (RBM5 overexpression) cells were fewer than 40% of that in control group. To certain extent, the migration of cells may be influenced by cell proliferation. But on the whole, our data suggested that RBM5 suppressed both migration and invasion of human gliomas cells.

### RBM5 suppressed the Wnt/β-catenin signaling and induced cell apoptosis

**Fig. 5:**
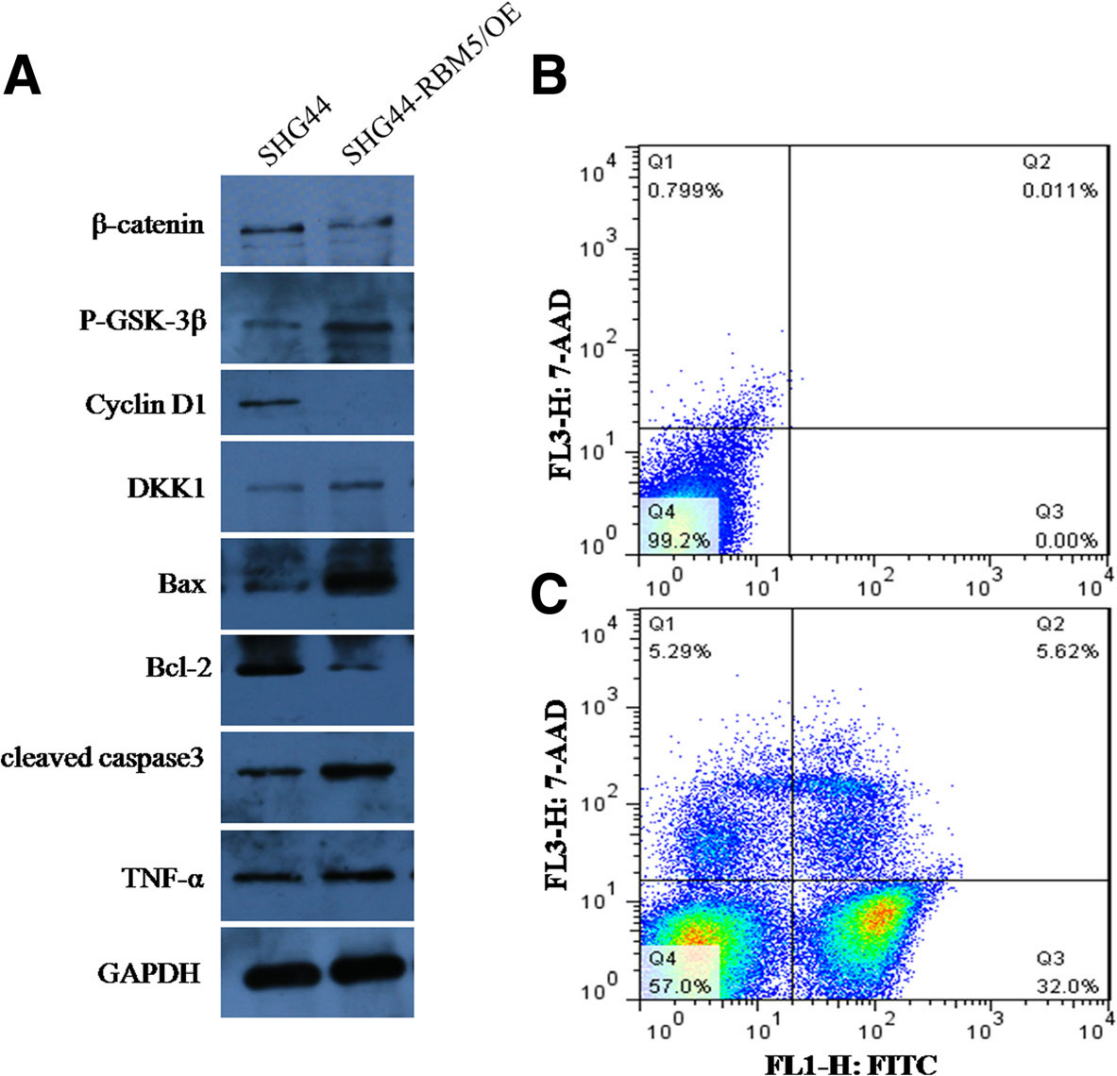
RBM5 inactivated Wnt/β-catenin signaling and induced apoptosis in SHG44 cells. Proteins were extracted from SHG44 cells with or without RBM5 overexpression. **a** Expression of β-catenin, P-GSK-3β, Cyclin D1, DKK1, Bax, Bcl-2, cleaved caspase3, and TNF-α were determined by western blot. GAPDH was used internal control. **b** FACS analysis of the apoptotic rate of SHG44 cells without RBM5 overexpression. **c** FACS analysis of the apoptotic rate of SHG44 cells with RBM5 overexpression. All experiments were repeated in triplicates. *P < 0.05 showed the significant difference

RBM5 was most frequently reported to induce apoptosis and cell G1/S arrest [12]. Also, it was shown to inhibit canonical Wnt/β-catenin signaling [14]. So, we determined the critical molecules in both apoptosis and Wnt/β-catenin signaling pathway. In consistent with the previous report, Wnt/β-catenin signaling was blocked by RBM5. The typical molecules including β-catenin and Cyclin D1 were significantly decreased in SHG44-RBM5/OE cells. On contrary, P-GSK-3β and DKK1 levels were increased (Fig. 5a). Meanwhile, the apoptosis signaling was activated by RBM5. As shown in Fig. 5a, Bcl-2 expression was reduced greatly while pro-apoptotic molecules including Bax, TNF-α, and cleaved caspase3 were upregulated remarkably in SHG44-RBM5/OE cells. And the apoptotic rate in SHG44-RBM5/OE cells increased greatly compared to the control (from 0 to 32%). So, our data suggest that RBM5 could simultaneously inhibit Wnt/β-catenin signaling and activate apoptosis in human gliomas cells.

## Discussions

Gliomas, one of the most common malignant brain tumors, showed increasing morbidity and mortality in recent years. But regardless of the unsatisfactory prognosis of gliomas, elucidation of implicit molecular mechanism have enriched our knowledge about tumor and pushed forward the war against tumor. Of the reported critical genes in tumors, RBM5 was a tumor suppressor and enhanced tumor cell apoptosis in a list of tumors. Peng et al. demonstrated that RBM5 underexpression was closely correlated with poor clinicopathological features in pancreatic cancer [16]. Consistently, RBM5 was shown to be weakly expressed in either gliomas tissues or cell lines here. And RBM5 expression was clinically correlated with tumor stage but not sex. This was inconsistent with the previous study in which gliomas incidence was reported higher in male patients than that in female patients aged between 30 and 40 years [17]. Maybe a larger cohort of gliomas patients is necessary to support this conclusion. In addition, patient with low RBM5 expression showed worse prognosis than those with high RBM5 expression. These data pointed out the clinical value of RBM5 in gliomas.

To investigate the role of RBM5 in gliomas, we successfully constructed a gliomas cell line stably expressing RBM5 with lentivirus vector. Our data demonstrated that upregulated RBM5 significantly inhibited cell growth and disrupted DNA synthesis in gliomas cells. DNA synthesis is a critical process in cell cycle and keeps the maintenance of genetic information during mitosis which is ensued by proliferation. Further, both migration ability and invasive capacity of gliomas cells were markedly suppressed by RBM5 overexpression. Migration and invasiveness generally precede metastasis during tumor progression. And it is known that tumors are characterized by uncontrolled cell proliferation and potent aggressive ability [18]. Based on these facts, we concluded that RBM5 played a suppressor role in gliomas.

But how did RBM5 suppress oncogenesis and metastasis in gliomas? The first as well as the most reported mechanism was that RBM5 could activate apoptotic signaling and enhance cell apoptosis [12]. Apoptosis, also called programmed cell death (PCD), was a complex activity involving multiple genes and was vital to the physiological and pathological process of cell life. Bcl-2 and Bax, often form heterodimers, are two most important regulators in apoptotic signaling pathway [12]. They are reversely correlated during PCD process which means that Bcl-2 antagonizes the effect of Bax and vice verse. Caspase cascade response directly caused cell PCD, and this process includes sequentially activation of key effectors such as caspase-9 and caspase-3. In lung cancer cells transfected with RBM5, pro-apoptotic protein Bax was increased while anti-apoptotic protein Bcl-2 was decreased reversely [12]. Meanwhile, mitochondrial apoptotic pathway was also activated by RBM5 as caspase-9 and caspase-3 activity was upregulated greatly. In another study, expression of cleaved caspase-9 and cleaved caspase-3 was upregulated by RBM5 in prostate cancer [10]. In this study, we demonstrated that Bcl-2 was downregulated while the level of Bax, cleaved caspase-3, and TNF-α was increased significantly by RBM5 in gliomas cells, which suggest that RBM5, at least partly, promoted cell apoptosis in gliomas cells. And apoptosis assay further confirmed the apoptosis-inducing function of RBM5. The second mechanism of RBM5 involves its anti-proliferative effect and cell cycle arrest. RBM5 was reported to inhibit cell growth and colony formation through increased p53 in H1299 cells while caused cell cycle arrest at G1 phase [19]. At the same time, RBM5 was shown to antagonistically regulate the proliferative ability of cancer cells through alternative splicing of NUMB gene [8]. However, different with the early reports, we found in this study that Wnt/β-catenin signaling was activated by RBM5 in gliomas cells. Wnt/β-catenin signaling was one typical proliferation-promoting signaling pathway and has been shown to be activated in various kinds of tumors. Besides the above two mechanisms, other signaling may be also involved in the anti-tumor role of RBM5. For example, RBM5 was also shown to downregulate EGFR expression and induce cell autophagy [20, 21]. Autophagy is another autonomic programmed cell death or type II PCD. But its role in gliomas needs more study. Accordingly, our data proved that RBM5 promoted tumorigenesis of gliomas through activation of Wnt/β-catenin signaling and increased cell apoptosis.

## Conclusions

In summary, RBM5 was downregulated in gliomas and disrupted both proliferation and migration ability of gliomas cells by activating Wnt/β-catenin signaling and inducing cell apoptosis. Our data suggest that RBM5 plays a suppressor role in gliomas and provide a new promising therapeutic target for patients with gliomas.

## Abbreviations

Bcl-2: B cell lymphoma-2
FACS: Fluorescence-activated cell sorting
MTT: 3-(4,5-dimethylthia-zol-2-yl)-2,5-diphenyltetrazolium bromide
NSCLC: Non-small cell lung cancer
PCD: Programmed cell death
P-GSK-3β: Phosphorylated GSK3β
RBM5: RNA-binding motif protein 5
RT-qPCR: Real-time quantitative PCR
TNF-α: Tumor necrosis factor-α
VEGF: Vascular endothelial growth factor
